# Evaluation of imaging findings in gastrointestinal anisakiasis in emergency CT and ultrasound

**DOI:** 10.1186/s13244-023-01511-9

**Published:** 2023-11-13

**Authors:** Roberto Fornell-Perez, Maite Urizar-Gorosarri, Uxue Martinez-Urabayen, Marta Perez-Bea

**Affiliations:** Radiology Department, Basurto Universitary Hospital, Bilbao, Vizcaya Spain

**Keywords:** Anisakis larvae, Gastric anisakiasis, Intestinal anisakiasis, Computed tomography, Ultrasonography

## Abstract

**Background:**

To assess the frequency of appearance of various signs (isolated and grouped) in emergency imaging tests in patients with anisakiasis, according to the location of gastrointestinal tract involvement.

**Methods:**

Retrospective review by two experienced radiologists of emergency ultrasounds and CTs performed on patients admitted in the Emergency Department of our hospital with later confirmed anisakiasis (2010–2021), assessing the presence of signs suggesting anisakiasis. Calculation of the frequency of appearance according to the gastric or intestinal location, as well as the most common grouped signs.

**Results:**

Out of 231 total patients with anisakiasis, imaging studies were performed in 144: abdominopelvic ultrasound in 43 cases and CT in 111 (both techniques in 31). In cases with gastric occurrence (34), in CT the wall stratification (100%), wall thickening (97%), fat stranding (91%) and ascitic fluid (82%) were predominant. In the intestinal cases (105), in CT (95) the wall thickening (100%), fat stranding (92%) and mesenteric vessel engorgement (83%) were usual; in ultrasound (40), ascitic fluid and wall thickening (70% in both cases) were frequently observed. The frequency of grouped appearance of the mentioned signs was 82% in gastric cases, 80% in intestinal cases and 50% in ultrasounds. Multisegment involvement in CT reached 28% (gastric + intestinal) and 11% (only intestinal) of cases.

**Conclusions:**

The most frequent CT findings in patients with gastric anisakiasis are wall stratification and thickening, fat stranding and ascitic fluid. In the intestinal cases, wall thickening, fat oedema and vessel engorgement are the most often observed findings.

**Critical relevance statement:**

The presence of different radiological signs makes it advisable to include anisakiasis in the differential diagnosis of acute abdomen. Intestinal and multifocal involvement rates are greater than previously reported.

**Key points:**

• In gastric anisakiasis, CT frequently shows wall stratification and thickening, fat stranding and ascitic fluid.

• In intestinal anisakiasis, CT often presents wall thickening, fat stranding and vessel engorgement.

• In intestinal anisakiasis, ultrasounds most frequently show ascitic fluid and wall thickening.

**Graphical Abstract:**

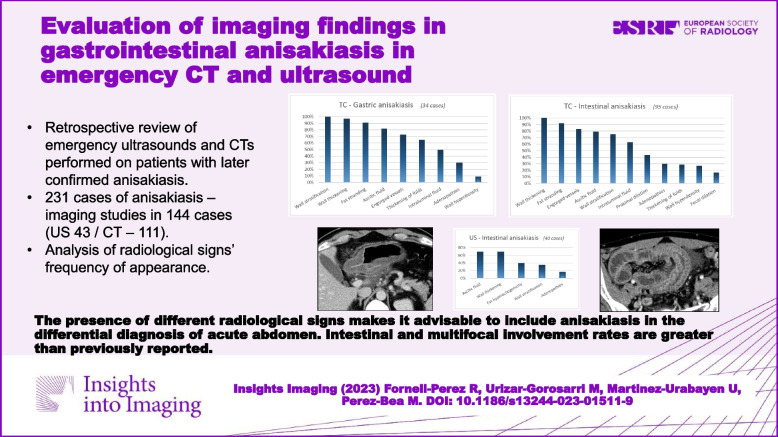

## Background

Anisakiasis is a parasitosis with principally gastrointestinal involvement, associated with the ingesting of anisakis nematode larvae in undercooked fish or seafood [1, 2]. A high percentage of cases occur in Japan (more than 90% of the approximately 20,000 yearly cases), probably in relation to dietetic habits; however, a rising trend has been observed in other regions such as Europe and North America [1–3].

The symptoms are non-specific, usually debuting as an acute abdominal condition of variable severity, on occasion associated with allergic symptoms [1, 2]. The latency period of the ingestion of parasites and clinical manifestations is variable, depending on the affected area of the digestive tract [2, 4]. Although the interval in gastric involvement is usually less than 24 h, the interval in the intestinal cases is usually greater (up to 1 week), which makes the association between the emergency conditions and the high-risk food difficult in carrying out the anamnesis [1, 4]. The diagnostic suspicion is oriented towards more common inflammatory conditions such as appendicitis, cholecystitis or diverticulitis [1, 5].

**Fig. 1:**
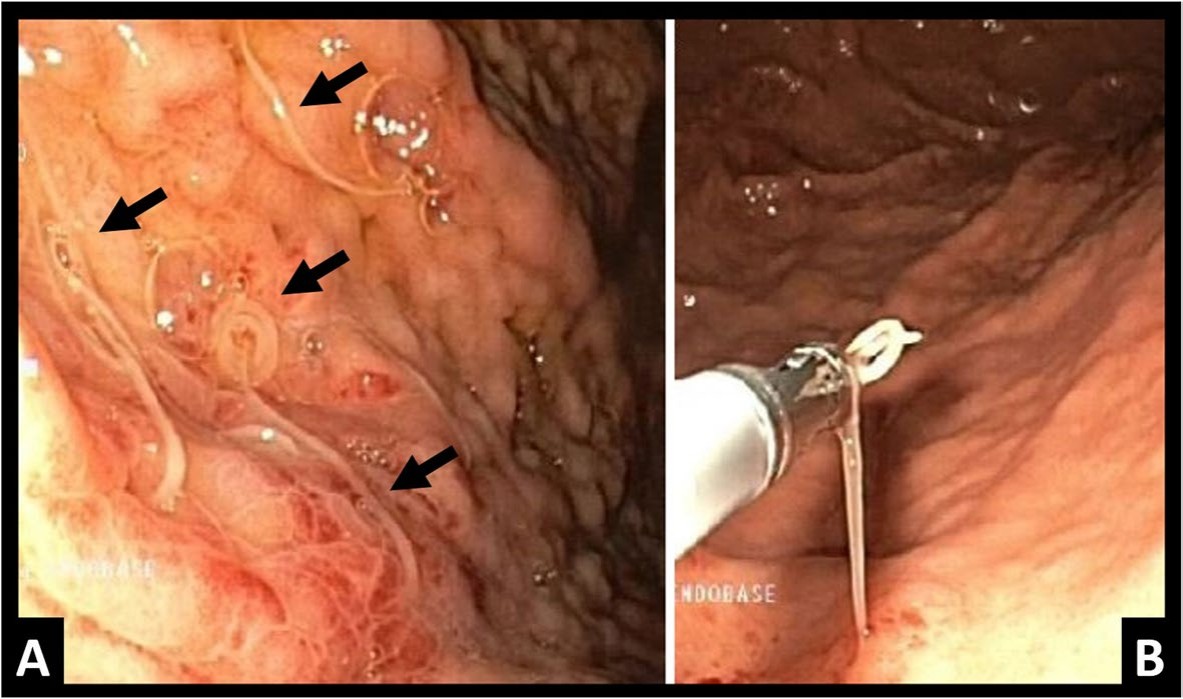
Programmed gastroscopy of two patients during hospital admission. Direct visualisation of Anisakis, in the first case, multiple (**A**, black arrows) and in the second, showing the extraction (**B**)

Diagnostic certainty is provided by direct observation of the parasites in endoscopic tests or in surgery (Fig. 1) [2, 6, 7]. However, in the context of a non-specific acute abdominal condition, it is frequent to turn to assessment by means of imaging tests. The posing of possible anisakiasis through radiological findings could be key in guiding the clinician in unsuspected cases, mainly in intestinal cases, given the non-specific clinical manifestations and the longer time transpired from contamination [1, 4, 8].

There are few studies that analyse the radiological findings associated with anisakiasis, which have limited case series and assess different signs. In addition, all computed tomography (CT) and most ultrasound series correspond to Asian countries; however, there are indications of epidemiological differences between regions, with a higher number of intestinal cases in Europe with respect to the marked gastric predominance in Japan [2, 9]. Is it possible that there are also differences in the radiological manifestations, secondary to the epidemiological variations?

The objective of our retrospective study was to analyse the frequency of appearance of a wide spectrum of radiological signs in emergency CTs and ultrasounds (isolated and grouped), differentiating between gastric and intestinal location, in a sample of patients admitted in the Emergency Department of our centre due to an acute abdominal condition with definitive diagnosis of anisakiasis.

## Methods

The research project was carried out according to the ethical standards established by the Declaration of Helsinki, with the approval of the Clinical Research Ethics Committee of our centre. Informed consent was waived because of the retrospective nature of the study.

### Patients

A record of patients admitted in the Emergency Service and with a definitive diagnosis of anisakiasis in the period between January 2010 and December 2021 was requested from the Clinical Documentation Service of our centre. Of these patients, only the cases which had undergone imaging tests (either conventional radiology, abdominopelvic ultrasound and/or CT) during their stay were selected. In contrast to CT and ultrasound, results of conventional abdominal radiographs were only regarded as secondary findings.

The inclusion criteria were (a) definitive diagnosis of anisakiasis after the acute abdominal process, confirmed by endoscopy or antigen test [10]; (b) performance of at least one imaging test during the first 48 h after admittance in the Emergency Service; (c) radiological images available for review; and (d) in case of performance of a CT, availability of at least one portal venous phase after administering intravenous contrast.

As exclusion criteria, the following were considered: (a) absence of confirmed diagnosis of anisakiasis; (b) technically deficient radiological images; and (c) background of liver disease, serious cardiovascular disease, Crohn’s disease, or an active tumoral disease in the patients’ clinical records. This last criterion was included in order to avoid a possible bias of confusion secondary to the overlapping of some signs in imaging.

A radiologist in training compiled data on the sample of patients from their clinical history: demographical data, symptoms from the emergency clinical condition, clinical suspicion, history precedent of food exposure, timing to the onset of symptoms, complementary tests, later endoscopic findings and complications in case they existed.

### Images acquisition

All the radiological studies were made in a single centre using the same equipment. The abdominopelvic ultrasounds (Epiq 5, Philips) were done by the on-call radiologist with a convex 5 MHz probe for general assessment and linear 12 MHz probe for the intestinal assessment in case of observing pathological segments. For the CTs, equipment with 128 detectors (Somatom Definition AS, Siemens) was used; in all cases, the same protocol was applied, with the acquisition of the study 70 s after the administering 70 ml of intravenous iodine-based contrast (Iomeprol 400 mg/ml) at 2 ml/s through the antecubital vein (axial reconstruction with a slide thickness of 3 mm; 1 mm thickness available for coronal or sagittal reconstruction on demand); in some cases, depending on the initial clinical suspicion, additional phases were acquired.

### Interpretation of images

Two radiologists with prior experience in abdominal radiology (10 and 6 years) independently reviewed the images of each study (conventional radiology, ultrasound and CT) according to previously established criteria. Since all the patients had a confirmed diagnosis of anisakiasis as an inclusion criterion, the reviewers were not blind to this information. In the conventional radiology, the presence of focal or generalised dilation of the stomach and/or the bowel loops was assessed. In both CT and abdominopelvic ultrasound, the presence of signs that have been related to anisakiasis in the literature in the affected gastrointestinal segments was assessed [4, 5, 11–13]: wall thickening or of folds/valves; presence of wall hyperdensity (CT) or stratification (CT or ultrasound); ascitic fluid (local or generalised); perienteric fat stranding (CT) or hyperechogenicity of mesenteric fat (ultrasound); engorgement of the adjacent mesenteric vessels (CT); and regional adenopathies. Bowel dilation and its degree (calibre in mm), intraluminal fluid and associated complication signs (obstruction, pneumoperitoneum, etc.) were also assessed. In case of doubt, a conclusion was reached through consensus.

The digestive wall thickness was assessed at the maximum visualised point that was reliably measurable; the intestinal wall over 3 mm was considered thickened, and the gastric wall over 10 mm in case of existing content [14, 15]. Folds thickening has been described as a gastric finding in endoscopic studies, as well as oedema of the valvulae conniventes in ultrasound of intestinal anisakiasis [2, 5]; since there is not a previously established measure, the sign was considered positive when prominent oedematous folds were observed in the lumen. Measurable folds with a thickness ≤ 5 mm were considered normal [16]. The degree of the fat stranding was assessed qualitatively in three degrees (mild, moderate or extensive), previously agreed by the reviewers. The presence of mural stratification or target appearance with a middle/outer low-density layer presumably represented the submucosal oedema. Engorged vessels were considered when prominent, thickened vessels were observed adjacent to the involved segment. Data were collected of the increase of the calibre of the small intestine loops over 25 mm, considering one calibre of the lumen ≥ 30 mm as significant dilation. Data were also compiled on the distribution of the ascitic fluid (focal point adjacent to the affected segment or generalised ascites). As for the adenopathies, the diameter in the short axis of the visible ganglia situated in the area of the affected segment was measured; those that surpassed the short axis diameter by >9 mm in the gastric area and by >5 mm in the mesenteric areas were considered [17, 18].

### Statistical analysis

For the statistical analysis, frequency calculations were made for each sign according to the technique and the affected segment (stomach or intestine, including both small bowel and colon). Then frequency calculations were made of the appearance of the most frequent signs grouped in different sets (between two and four signs), again according to technique and affected segment.

## Results

### Demographic data and generic results

Between January 2010 and December 2021, 231 cases of acute abdominal condition with admittance in our centre’s Emergency Service were finally diagnosed as gastrointestinal anisakiasis.

**Fig. 2:**
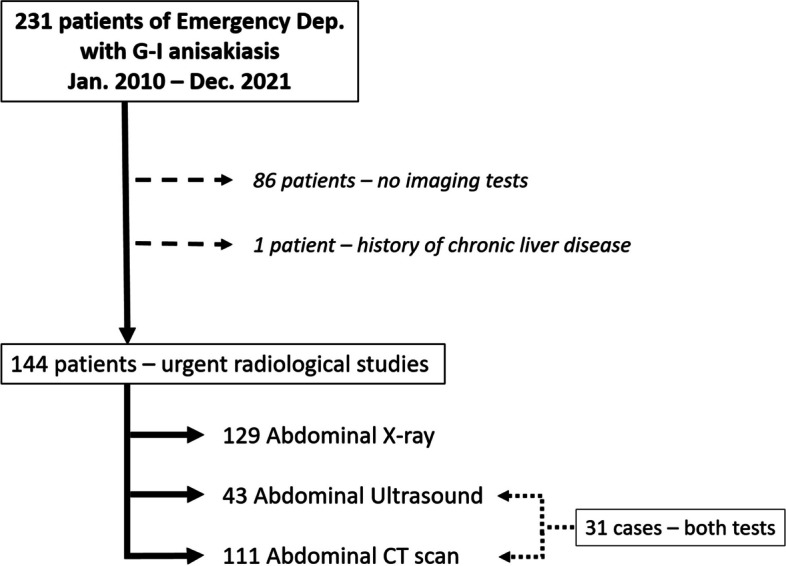
Workflow outline

The workflow is illustrated in Fig. 2. Of the total cases (231), emergency radiological studies were made in 145 patients; one of the cases was excluded due to presenting chronic liver disease with previous episodes of decompensation. Of the group of patients included with radiological studies, 75 were females and 69 were males, with a mean age of 53.69 years (ranging from 15 to 94, median 53).

In the group of the 144 patients that were finally included, a simple abdominal X-ray was performed on 129 of them, abdominopelvic ultrasound on 43 and abdominopelvic CT on 111. 108 patients had either abdominal plain films and ultrasound (37) or abdominal plain films and CT (96), while 21 patients had abdominal plain films as the only imaging test. Both ultrasound and CT were performed on 31 of the cases whereas 25 patients received all three imaging tests.

As for the endoscopic studies, gastroscopy was performed 1 to 5 days after admittance in 9 cases of gastric involvement (2 with visible parasites; 7 with mild inflammatory changes; mean 2.66 days), and 5 cases with exclusively intestinal involvement through imaging (3 with mild gastric inflammatory changes, 2 normal: ranging from 0 to 6 days, mean 2.4 days). In another 6 cases, a colonoscopy was performed (2 with mild inflammatory changes, 4 normal; ranging from 2 to 5 days, mean 3.66 days).

Surgery was chosen in only one case due to obstruction of undetermined cause, observing erythematous petechial spotting in the ileum as the only finding.

### Cases with abdominal plain films

Of the 129 patients with conventional radiology, focal dilation of loops could be observed in 47 cases and generalised dilation in 5 cases, 4 of them with signs suggesting intestinal obstruction.

In the cases with abdominal plain films only, a working diagnosis from the emergency room team has not been documented in the medical files.

### Cases with ultrasound and/or CT

In 123 patients, an ultrasound and/or CT was performed (43 and 111, respectively). The mean time that transpired between exposure to suspicious food and the appearance of symptoms was 26.96 h (minimum 1, maximum 168 h). Considering the cases separately according to the location of affected segments in imaging tests, the gastric cases presented a mean interval from ingestion of 21.7 h (ranging from 1 to 96 h), and the intestinal cases 24.5 h (ranging from 1 to 168 h); in the cases with exclusively intestinal involvement, the mean was 26 h. Exposure was documented with an undetermined interval in 13 patients (1 gastric, 2 gastrointestinal and 10 intestinal). There was no record of recent exposure to suspicious food in the medical files of 10 cases, all of them with exclusively intestinal involvement in imaging tests.

The clinical suspicions when requesting radiological tests were diverse: non-specific abdominal pain (54 cases), acute appendicitis (25) biliar pathology (14), diverticulitis (12), complication of anisakiasis (10), intestinal obstruction (5), aortic pathology (1), perforation (1) and ischemic colitis (1). In nine patients the coexistence of allergy symptoms with the abdominal condition was noted, principally cutaneous pruritus (8 cases), erythema in palms and soles (4) and exanthema (3 in axillae and groin, 3 facial and cervical).

The distribution of the involvement in the different gastrointestinal segments is shown in Table 1, with 34 cases of gastric anisakiasis and 105 intestinal anisakiasis out of the total 123 patients (22 of those patients with multisegment involvement in both stomach and intestine). The remaining 6 patients with verified anisakiasis did not show any gastrointestinal findings of interest in radiological studies (2 cases with ultrasound, 3 with CT and 1 with both techniques).

**Table 1:** Distribution of gastric and intestinal involvement due to anisakiasis visible in abdominopelvic ultrasound and CT (single or multi-segment involvement). Out of the total 123 cases with ultrasound and/or CT, 6 patients did not show any radiological finding (2 US cases, 3 CT cases and 1 with both US and CT)

	**Nº of cases**
**Stomach**	34
Gastric body	12
Gastric antrum	34
**Intestine**	105
Duodenum	9
Jejunum	11
Ileum	80
Right colon	22
Left colon	3
**Multisegment involvement**	35
*Gastric + intestinal*	22
G + I	10
G + D	3
G + J	3
G + RC	3
G + J + I	1
G + I + RC	1
G + J + I + RC	1
*Intestinal − multisegment*	13
I + RC	9
I + J	2
I + LC	1
I + RC + J	1

*G*, stomach; *D*, duodenum; *J*, jejunum; *I*, ileum; *RC*, right colon; *LC*, left colon

Of the total 111 CTs and 43 ultrasounds, pathological findings were observed in 107 and 37 of them, respectively. The list of signs in CT related to the presence of anisakis on the gastric and intestinal levels are shown in Tables 2 and 3, respectively. Moreover, Table 4 shows the ultrasound signs in those cases that showed intestinal involvement.

**Table 2:** CT signs of gastric anisakiasis (total 34 patients)

**Gastric anisakiasis (CT)**	**Nº of cases**	**%**
Wall stratification	34	100
Wall thickening	33	97
Fat stranding	31	91.1
*Mild*	7	20.6
*Moderate*	16	47.0
*Extensive*	8	23.5
Ascitic fluid	28	82.3
*Generalised ascites*	25	73.5
*Local adjacent fluid*	23	67.6
Engorged mesenteric vessels	25	73.5
Thickening of folds	22	64.7
Intraluminal fluid	17	50
Regional adenopathies	10	29.4
Wall hyperdensity	3	8.8

**Table 3:** CT signs of intestinal anisakiasis (small bowel and colon; total 95 patients)

**Intestinal anisakiasis (CT)**	**Nº of cases**	**%**
Wall thickening	95	100
Fat stranding	88	92.6
* Mild*	35	36.8
* Moderate*	35	36.8
* Extensive*	18	18.9
Engorged mesenteric vessels	79	83.1
Ascitic fluid	75	78.9
* Local adjacent fluid*	68	71.5
* Generalised ascites*	59	62.1
Wall stratification	71	74.7
Intraluminal fluid	60	63.1
Proximal dilation	41	43.1
Regional adenopathies	29	30.5
Thickening of folds	28	29.4
Wall hyperdensity	26	27.3
Focal dilation of the lumen	16	16.8

**Table 4:** Ultrasound signs of intestinal anisakiasis (total 40 of 43 patients)

**Intestinal anisakiasis (US)**^a^	**Nº of cases**	**%**
Ascitic fluid	28	70
*Generalised ascites*	23	57.5
*Local adjacent fluid*	17	42.5
Wall thickening	28	70
Hyperechogenicity of mesenteric fat	16	40
Wall stratification	14	35
Regional adenopathies	7	17.5

^a^Of the total 43 US cases, 2 presented no defined radiological location and 1 had exclusive gastric anisakiasis (not included in this table)

Regarding the 31 patients in whom both CT and ultrasound were performed, both tests coincided in detecting ascitic fluid in 19 patients (1 more case was only seen on ultrasound and 3 cases only on CT), wall thickening in 16 and fat alterations in 8 (13 and 4 more cases only on CT, not detected on ultrasound, respectively). In six patients with signs of gastric involvement in the CT, an ultrasound was performed previously, observing ascites as the only relevant finding in four cases (66.67%); however, only one of this group presented gastric pathology exclusively, thus three cases with ascites presented concomitant intestinal involvement.

**Fig. 3:**
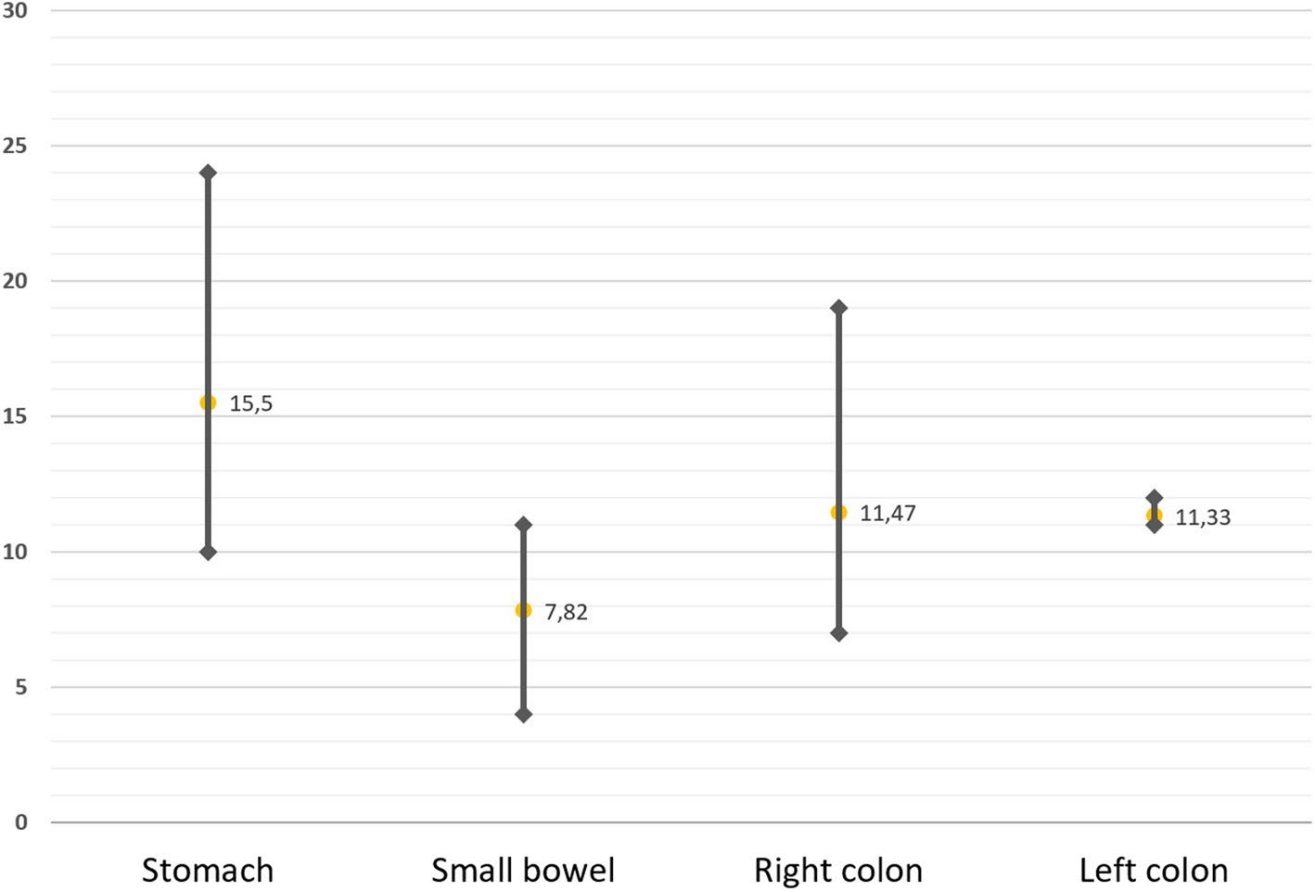
Ranges of wall thickening and mean value in centimetres according to the affected segment of the digestive tract

The cases of intestinal wall thickening in ultrasound presented a mean thickening of 4.9 mm (ranging from >3 to 8 mm, median 4.5 mm). In CT, differences in the range of thickening were observed according to the segment involved (Fig. 3).

In 31 cases significant dilation of small intestine loops adjacent to the affected segment was observed (calibre: ranging from 30 to 38 mm, average 31.9 mm, median 31 mm), in 27 cases, proximal segmental and in 4 cases, extensive dilation (dilation of multiple proximal loops, affecting a large segment with obstruction criteria). In another 10 cases, non-pathological focal dilation was observed (25–29 mm). No dilation in the pathological range of the colon was observed in any case.

Five cases of suspected complications secondary to anisakiasis were observed through imaging (four of an obstructive condition and one colo-colonic intussusception), all of these patients with intestinal involvement and with resolution with conservative treatment.

The frequency of grouped appearance of the signs most usually detected is shown in Table 5, broken down by technique and affected portion.

**Table 5:** Frequency of appearance of different groups of the most frequent signs, according to technique and digestive location. In each group, the different levels show the subsets created by adding signs of lower frequency

**Frequency of grouped appearance of signs**	**Nº of cases**	**%**
***CT signs − gastric (34 cases)***		
Wall stratification and wall thickening	33	97
Wall stratification and wall thickening **+** fat stranding	31	91.2
Wall stratification and wall thickening **+** fat stranding** +** ascitic fluid	28	82.3
Wall stratification and wall thickening **+** fat stranding **+** engorged mesenteric vessels	25	73.5
***CT signs − intestinal (95 cases)***		
Wall thickening and fat stranding	88	92.6
Wall thickening and fat stranding **+** engorged mesenteric vessels	76	80
Wall thickening and fat stranding **+** ascitic fluid	73	76.8
Wall thickening and fat stranding **+** wall stratification	65	68.4
***Ultrasound signs − intestinal (40 cases)***		
Wall thickening and ascitic fluid	20	50
Wall thickening and ascitic fluid **+** hyperechogenicity of mesenteric fat	13	32.5

## Discussion

**Fig. 4:**
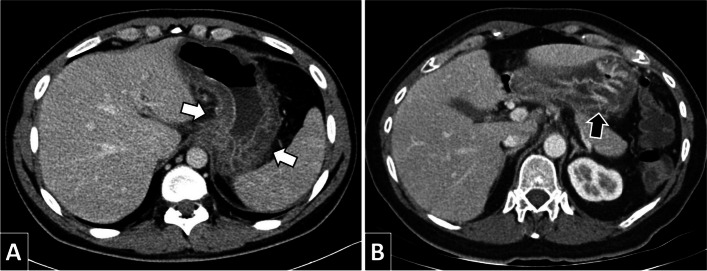
Patients of 31 (**A**) and 50 years (**B**) of age with gastric anisakiasis by CT. The diffuse thickening of the gastric wall with marked submucosal oedema (**A**, white arrows), as well as the oedema of the folds (**B**, black arrow) can be observed

**Fig. 5:**
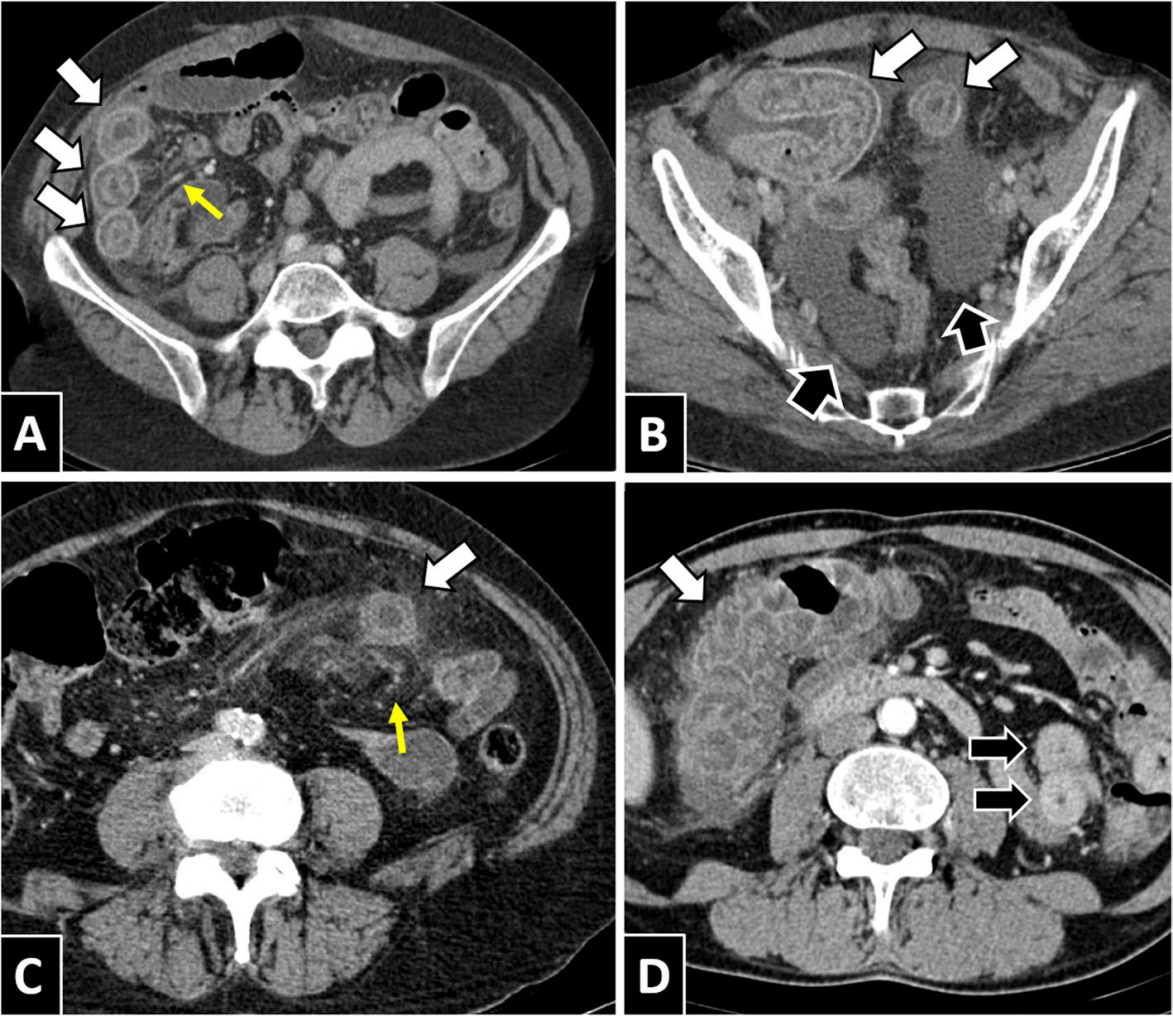
Intestinal involvement due to anisakis in different locations. **A** and **B** Patient of 58 years of age with ileal anisakiasis, showing wall thickening, target appearance suggestive of submucosal oedema (white arrows), engorged mesenteric vessels (yellow arrows) and ascitic fluid (black arrows). **C** Patient of 68 years of age with similar findings and in addition oedema of the adjacent mesenteric fat. **D** Patient of 54 years of age with anisakiasis in various segments. The important involvement of the right colon (white arrows) can be noted, which showed wall and folds thickening, wall stratification, engorged mesenteric vessels and slight fat stranding. On the other hand, it also presented hyperdense circumferential wall thickening and slight engorgement of mesenteric vessels in a long segment of the ileum (black arrows)

In this study, we have conducted an analysis of the frequency of appearance of different radiological signs in relation to the diagnosis of gastrointestinal anisakiasis, observed in emergency studies of patients with an acute abdominal condition. In the cases of gastric involvement, in CT in decreasing order, wall stratification, wall thickening, perienteric fat stranding and ascitic fluid are predominant (Fig. 4). On the other hand, in the intestinal cases, the wall thickening, fat stranding, engorgement of the adjacent mesenteric vessels and ascitic fluid were more frequent (Fig. 5). Furthermore, the ultrasound in intestinal cases showed with greater frequency ascitic fluid and wall thickening. As far as we know, it is the largest series of radiological cases and signs analysed in this pathology [4, 5, 11–13, 19].

The major problem for the diagnosis of anisakiasis through radiology is the non-specificity of the associated signs, which are shared with many inflammatory and infectious pathologies [1, 19]. However, our results show that certain sets of signs can be observed frequently in these cases (more uniformly in gastric involvement). Posing it as a diagnostic possibility in an appropriate context can be of great use for the clinician, given that it is not a usual suspicion, and a directed anamnesis is necessary to assess the exposure to risk: the radiological findings can be the first indication [4].

From a clinical point of view, reaching a definitive diagnosis can be especially challenging in unsuspected or intestinal cases [20]. The classic confirmation of anisakiasis is the direct visualisation of parasites, either in endoscopic studies, surgery, or emesis. The detection of high levels of anti-A. simplex IgE in serological tests presents high sensitivity (70 to 100%) and specificity (50 to 100%) [1, 20]; however, the interpretation can be complex due to the possibility of cross-reactions with other parasites or appearance in asymptomatic people who frequently consume fish [1, 2]. It has been suggested that the diagnosis of anisakiasis can be made in cases that meet certain criteria: (1) abdominal pain and/or allergic reaction after recent exposure to fish, (2) lack of reaction to fish proteins on skin testing; (3) elevated specific anti-A. simplex IgE or anisakis IgA/IgG levels in serological tests; (4) in intestinal cases, those findings can be associated with segmental intestinal oedema and proximal dilation of small bowel on CT scan [1, 3].

In the literature, there are few series of radiological cases on gastrointestinal anisakiasis; in general, they refer to CT findings. Shibata et al. [4] carried out a review of multiple signs in 41 cases, although without including wall thickening; it is the only series that assessed the presence of adenopathies, as far as we know. Kim et al. [11] assessed wall thickening and ascites in 30 cases with CT amid a clinical series. In addition, Takabayashi et al. [12] made a similar assessment in a larger-sized series, including the fat stranding, and Lee et al. [13] also included submucosal oedema to these last signs, but in a small series of exclusively intestinal cases.

Previous clinical series have documented a marked predominance of gastric cases [1, 9, 21]. Furthermore, the radiological series coincide in a distribution in a similar number of gastric and intestinal cases (47 to 50%, minimal intestinal predominance), unlike the clear predominance of intestinal involvement in our series [4, 11, 12]. These differences could be due to the method of sample selection; however, the previous radiological series present similar forms of case selection. A second possibility would be the influence of epidemiological aspects and of the diagnostic methodology according to geographic location, since nearly all the previous series correspond to Asian countries [2, 3, 5, 19]. It has been described that the intestinal involvement is more frequent in Europe than in Asia [3, 9]. There are different species within the *Anisakidae* family for which possible variations in associated symptoms have been described [2]; similarly, the parasitisation area or its radiological manifestations could also present certain differences. Furthermore, it has been posed that the occurrence of intestinal anisakiasis is probably greater than what is published, since the absence of definitive methods makes diagnosis difficult [11, 19, 22]. Another added factor would be the greater interval from ingestion in the intestinal cases, which complicates their relation to the acute condition; it can be observed in our series that most patients that do not mention a precedent correspond to this location.

Overall, our results are in line with the previous CT series, with certain exceptions. The first would be the presence of ascitic fluid (assessed overall): with respect to other publications, we have observed a slightly lower frequency of appearance in cases of intestinal involvement (78%, in previous articles 80–100%) and greater in gastric cases (82%, previous 33–70%). The high occurrence of multisegment involvement that we have observed in the cases of gastric involvement (22 of 34 patients) can be partly responsible for the difference on the gastric level. In addition, the appearance of submucosal oedema in the intestinal anisakiasis has also shown a lower frequency, which reached 100% of the patients in a previous study [4]. Also, in that series, a higher frequency of adenopathies was identified, especially in the intestinal cases (52%); this could be due to differences in the size ranges considered pathological, or, once again, to epidemiological factors.

The multisegment involvement has been described previously in the literature [23, 24]. We have not found data that assess its frequency in clinical series. According to our results, its occurrence could be significant (28.4%). Although we do not have definitive endoscopic confirmation of the involvement of both segments in any of these cases, the radiological findings are usually considered essential in the detection of intestinal involvement (Fig. 5d) [1, 25].

As for ultrasound findings, our data show differences with respect to the few available series. A greater frequency of ascites (87.5–91.6%) and of wall thickening (up to 100%) has been documented [5, 19, 26]. The stratified aspect of the wall with muco-submucosal oedema has been detected in a lower number of cases (up to 62%) [5].

In our opinion, this variation could be associated with differences in the patient selection methods: the previous series are usually small-sized and specifically oriented to cases of intestinal anisakiasis demonstrated by ultrasound. If we only consider the cases of our sample with wall thickening, results for ascites and wall stratification get closer to other studies (up to 72% and 50%, respectively). The inclusion in our series of patients with ultrasound without findings but pathology visible in CT makes the comparison difficult, but it could offer more extrapolative data. In addition, as far as we know, the presence of adenopathies or the oedema of mesenteric fat has not been assessed previously.

**Fig. 6:**
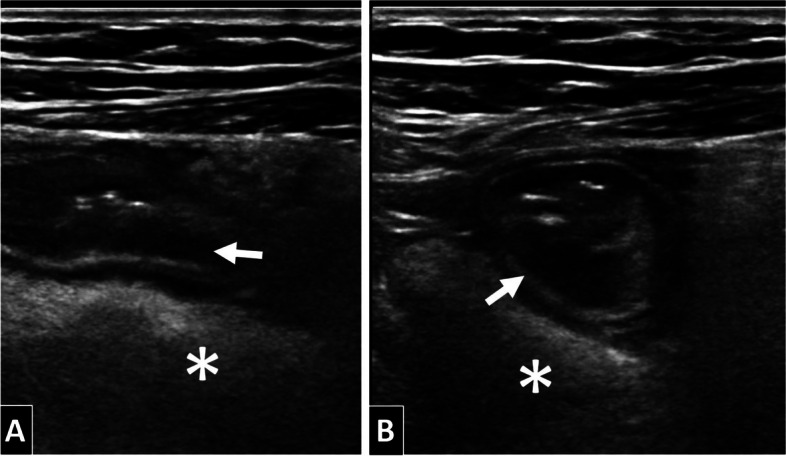
Patient of 62 years of age with ileal anisakiasis visible in ultrasound, sagittal (**A**) and axial view (**B**). The wall thickening can be observed due to oedema in the Kerckring valves (first hypoechogenic layer from the lumen, of submucosal location − white arrows), with important hyperechogenicity of the adjacent mesenteric fat (asterisks) due to severe oedema

It is noteworthy that the wall thickening due to the Kerckring valves (first hypoechogenic layer from the lumen, corresponding to the muscularis mucosae) with preservation of the stratification in layers seems to be a relatively characteristic finding (Fig. 6) [5, 19]. However, it is not specific, since it can be observed in other entities such as mesenteric venous thrombosis, congestive heart failure, angioedema, enteral lupus or radical enteritis [5, 19].

Our study presents various limitations. In the first place, its retrospective nature; a prospective study would be complex, given the low frequency of the pathology. In addition, the absence of a control group does not allow assessing parameters of sensitivity or specificity of the tests: although this information would be interesting, it was not the objective of our study, and the low occurrence would again be a problem. Secondly, the method of patient selection in our study could create a bias, given the predominance of more serious cases: on one hand, it was posed that many cases of anisakiasis (possible mild conditions or those with a predominance of allergy symptoms) do not go to the hospital and are not diagnosed [3, 7, 21]; on the other hand, it is reasonable to think that in the mild cases that do reach the emergency services, radiological studies are requested less frequently. In any case, the sample would correspond to the type of patient that usually requires imaging tests, for which reason we consider the results to be representative. In third place, the assessment of the specific origin of generalised ascites is limited in the cases with both gastric and intestinal involvement; which could introduce a bias in the assessment of this sign. Lastly, ultrasound is a highly operator-dependent technique, which may have not correctly pictured all the ultrasound findings; given the retrospective nature of the study, this is a limitation that could lead to underestimating the signs that have not been recorded.

## Conclusion

In an appropriate context, certain radiological findings can make the inclusion of anisakiasis be recommendable in our differential diagnosis given its frequent relation; sometimes these findings will be the first indication that orients the clinical suspicion. In CT, the appearance of wall stratification and thickening, perienteric fat stranding and ascitic fluid is very frequent in cases of gastric anisakiasis. In intestinal cases, wall thickening, fat stranding, engorgement of the adjacent mesenteric vessels and ascitic fluid through CT and ascitic fluid and wall thickening by ultrasound are habitual. Moreover, the multisegment involvement could be far more frequent than commonly supposed.
